# Clinical impact of Hypofractionated carbon ion radiotherapy on locally advanced hepatocellular carcinoma

**DOI:** 10.1186/s13014-020-01634-z

**Published:** 2020-08-14

**Authors:** Shintaro Shiba, Kei Shibuya, Masahiko Okamoto, Shohei Okazaki, Shuichiro Komatsu, Yoshiki Kubota, Takashi Nakano, Tatsuya Ohno

**Affiliations:** 1 Department of Radiation Oncology, Gunma University Graduate School of Medicine, 3-39-22, Showa-machi, Maebashi, Gunma 371-8511 Japan; 2 Gunma University Heavy Ion Medical Center, 3-39-22, Showa-machi, Maebashi, Gunma 371-8511 Japan

**Keywords:** Carbon ion radiotherapy, Hepatocellular carcinoma, Hypofractionation, Vascular invasion

## Abstract

**Background:**

Hepatocellular carcinoma (HCC) involving a major branch of the portal or hepatic vein is in a locally advanced stage and remains difficult to cure. This study aimed to evaluate the clinical effects of carbon ion radiotherapy (C-ion RT) in locally advanced HCC (LAHCC).

**Methods:**

The data of 11 consecutive patients with LAHCC who received C-ion RT were analyzed. The C-ion RT doses of 52.8 Gy (relative biological effectiveness [RBE]) and 60.0 Gy (RBE) were delivered in 4 fractions for standard cases, and the 60.0 Gy dose was delivered in 12 fractions for close-to-gastrointestinal-tract cases. Survival and local control probabilities were calculated using the Kaplan-Meier method.

**Results:**

The median follow-up duration after C-ion RT was 36.4 months. The median age at the time of registration for C-ion RT was 76 years. The median tumor size was 53 mm. The numbers of treatment-naive and recurrent HCC patients were 1 and 10, respectively. Direct invasion of the major branch of the portal vein, hepatic vein, or both portal and hepatic veins was observed in three, five, and three patients, respectively. The 3-year overall survival, local control, and progression-free survival rates were 64, 78, and 18%, respectively. No patient developed radiation-induced liver diseases or grade 3 or higher toxicities in the acute and late phases.

**Conclusions:**

C-ion RT showed favorable clinical outcomes with a high rate of local control and minimal toxicities in LAHCC. Our findings suggest that C-ion RT is a promising multidisciplinary treatment option in LAHCC.

## Background

Hepatocellular carcinoma (HCC) involving a major branch of the portal or hepatic vein occurs in a locally advanced stage. Although molecular targeted therapy is the standard treatment for locally advanced HCC (LAHCC), according to the European Association for the Study of the Liver and European Organization for Research and Treatment of Cancer practical guidelines [1], LAHCC treated with molecular targeted therapy alone has shown dismal prognosis [2–4]. Therefore, radiotherapy, transarterial chemoembolization (TACE), hepatic arterial infusion chemotherapy, and/or percutaneous radiofrequency ablation (RFA) were performed in LAHCC patients as an additional treatment. Recently, the report by Yoon et al. showed that TACE combined with X-ray RT improved the prognosis compared with molecular targeted therapy alone in a randomized controlled trial [5].

Carbon ion radiotherapy (C-ion RT) provides both, physical and biological advantages over X-ray RT, and several researchers have shown favorable clinical outcomes in HCC patients when they were treated with C-ion RT [6–9]. In the physical aspect, previous studies have demonstrated a dose distribution advantage, showing that a reduced dose was delivered to the liver using C-ion RT compared with those of stereotactic body RT (SBRT) and intensity-modulated RT (IMRT) [10, 11]. This was achieved owing to the physical nature of the C-ion RT procedure with distal tail-off due to the Bragg Peak and a sharp lateral penumbra [12]. Additionally, in the biological aspect, the C-ion beams have higher linear energy transfer than X-rays, and thus have superior cell-killing effect in radioresistant tumor cells such as hypoxic and cancer stem cells [13, 14]. Although there is lack of data on the clinical outcomes in patients with LAHCC treated with C-ion RT, these advantages of C-ion RT may contribute to the improved prognosis of multidisciplinary treatment of LAHCC. Hence, in the current study, we analyzed the treatment outcomes of C-ion RT in patients with LAHCC.

## Methods

### Patients

We reviewed the medical records of 124 patients treated with C-ion RT for HCC at the Gunma University Heavy Ion Medical Center (GHMC) between July 2011 and August 2018. Eleven consecutive patients met the following criteria: 1) HCC involving a major branch of the portal or hepatic vein confirmed by histology or typical hallmarks of HCC using imaging techniques of four-phase multidetector-row computed tomography (CT) or dynamic contrast-enhanced magnetic resonance imaging (MRI) (hypervascular in the arterial phase with washout in the portal venous or delayed phase); 2) no intrahepatic metastasis or distant metastasis; 3) no findings suggesting direct infiltration of the gastrointestinal (GI) tract; 4) performance status (PS) ≤ 2 by Eastern Cooperative Oncology Group classification; and 5) Child-Pugh classification A or B. The definitions of the portal or hepatic vein and the Barcelona Clinic Liver Cancer (BCLC) classifications [15, 16] were determined using CT, MRI, ultrasonography, and other modalities. The albumin-bilirubin grade, by combining serum albumin and bilirubin, was calculated to evaluate liver function in all patients [17]. In the current study, recurrent HCC treated with TACE and/or hepatic arterial infusion chemotherapy and/or RFA was included. The treatment protocol was reviewed and approved by the Gunma University Institutional Review Board, and all patients signed an informed consent form before the initiation of therapy.

### Carbon ion radiotherapy

A heavy-ion accelerator at the GHMC was used to generate C-ion beams, and beam energies of 290 MeV/u, 380 MeV/u, and 400 MeV/u were selected according to the depth of the tumor. Doses of C-ion RT were expressed in Gy (relative biological effectiveness [RBE]), defined as the physical dose multiplied by the RBE of the C-ions [18, 19].

Treatment-planning CT and contrast-enhanced CT images were merged to precisely delineate the gross tumor volume (GTV). The clinical target volume (CTV) was defined as GTV plus 5 mm in all directions and modified to include microscopic disease progression and to exclude the GI tract and portal vein. A fiducial marker was inserted near the tumor before CT acquisition. Marker motion was measured from four-dimensional CT images, and a margin was calculated from marker motion by the motion management procedure [20]. The planning target volume (PTV) was generated by adding the margin to the CTV.

**Fig. 1:**
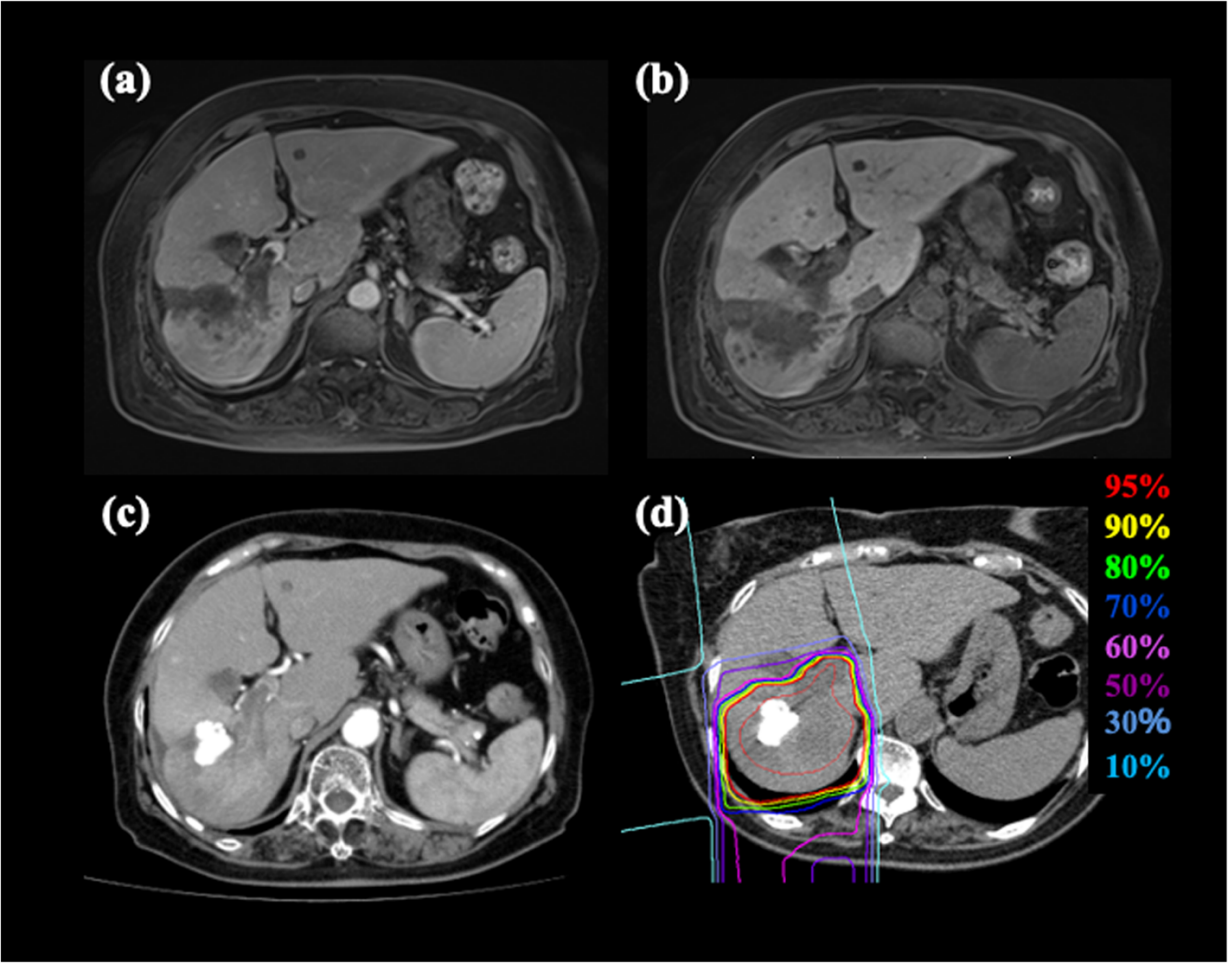
HCC in an 83-year-old female patient treated with C-ion RT. **a** MRI (early phase) before treatment. **b** MRI (late phase) before treatment. **c** CT (early phase) before treatment. **d** Dose distribution on axial CT images. Highlighted are: 95% (red), 90% (yellow), 80% (green), 70% (blue), 60% (pink), 50% (purple), 30% (light purple), and 10% (light blue) isodose curves (100% = 60 Gy [RBE]). The area within the red outline is GTV. CT, computed tomography; GTV, gross tumor volume; RBE, relative biological effectiveness

Prescribed doses were 52.8 Gy (RBE) or 60.0 Gy (RBE), delivered in four fractions for standard cases and the 60.0 Gy (RBE) delivered in 12 fractions for close-to-GI-tract cases. Close-to-GI-tract was defined as a distance of < 1 cm between the tumor and the GI tract. The planning aim was to cover the PTV with at least 95% of the prescribed dose. The dose constraints were as follows: D_1cc_ < 40 Gy (RBE) administered to the GI tract in the standard cases, D_1cc_ < 45 Gy (RBE) administered to the GI tract in the close-to-GI-tract cases, V_20_ < 35% administered to the liver, D_max_ < 52.8 Gy (RBE) administered outside the PTV at the porta hepatis (including the first branch of the portal vein and hepatic duct), D_max_ < 45 Gy (RBE) administered to skin in the standard cases, and D_max_ < 50 Gy (RBE) administered to skin in close-to-GI-tract cases [8]. Beam fields between 2 and 6 (median 2) were selected to satisfy the dose constraints for each patient. Figure 1 shows a representative case of the dose distribution and diagnostic imaging in LAHCC before C-ion RT.

For patient positioning, bone structural matching (BM) was basically performed on the orthogonal X-ray images [21]. The following positioning strategy was used according to the measured marker position from the BM position: BM was used if the absolute marker movement was less than 3 mm, with marker structural matching if the absolute movement was between 3 and 10 mm, and with a re-setup if the absolute movement was greater than 10 mm [22, 23]. After patient positioning, respiratory-gated irradiation was performed while monitoring the respiratory waveform detected from the respiratory sensor (Anzai Medical Co., Ltd., Tokyo, Japan).

### Evaluation during follow-up

Patients were followed up for 1 month after C-ion RT completion, and every 3 months thereafter. The follow-up examinations comprised a routine testing of blood cell counts and chemistry, and abdominal diagnostic imaging such as four-phase multidetector-row CT, dynamic contrast-enhanced MRI, or contrast-enhanced ultrasonography. Acute and late toxicities were graded according to the Common Terminology Criteria for Adverse Events, version 4.0, of the National Cancer Institute [24]. Liver toxicity was also assessed according to the changes in the Child–Pugh class. Acute and late toxicities were evaluated as the highest grade of toxicity that occurred within 3 months and at 3 months’ post initiation of C-ion RT, respectively. Local recurrence was defined as tumor regrowth, with contrast enhancement on CT, MRI, or ultrasonography in the irradiated field after C-ion RT.

### Statistical analyses

All statistical analyses were performed using the Statistical Package for the Social Sciences, version 25.0 (IBM Inc., Armonk, NY, USA). Survival was measured from the date of C-ion RT initiation to the date of death or the most recent follow-up. Local control (LC) was defined as no evidence of local progression. Progression-free survival (PFS) was measured from the initiation of C-ion RT to the date of local progression, disease progression outside of the primary site, or death from any cause. Probabilities of overall survival (OS), LC, and PFS rates were calculated using the Kaplan-Meier method. Additionally, we assessed the percentage of the minimum dose that covered 98% of the target volume (D_98_) based on the dose–volume histogram (DVH) for the CTV.

## Results

### Patient characteristics

The clinical characteristics of 11 eligible patients are summarized in Table 1. The median follow-up duration after C-ion RT was 36.4 (range: 4.3–86.2) months. The median patient age at the time of registration of C-ion RT was 76 (range: 57–86) years. The median tumor size was 53 (range: 27–119) mm. The numbers of treatment-naive and recurrent HCC patients were 1 and 10, respectively. The number of prior treatments of C-ion RT was one time in five patients, two times in three patients, eight times in one patient, and eleven times in one patient. In terms of prior treatment of C-ion RT for the target lesion, six patients had received TACE, two patients TACE and RFA, and two patients TACE and hepatic arterial infusion chemotherapy. Direct invasion in the major branch of the portal vein, the hepatic vein, or both portal and hepatic veins was observed in three (one with Vp4 and two with Vp3), five (one with Vv3 and four with Vv2), and three (all with Vp3 + Vv2) patients, respectively. Child-Pugh classes A and B were observed in 10 and 1 patients, respectively. The dose fractionation schedules were 52.8 Gy (RBE) in four fractions in two patients, 60 Gy (RBE) in four fractions in four patients, and 60 Gy (RBE) in 12 fractions in five patients.

**Table 1:** Patient characteristics (*N* = 11)

Characteristics	
Age, years, median (range)	76 (57–86)
Tumor size, mm, median (range)	53 (27–119)
Sex, number	
Male:Female	9:2
Etiology	
Hepatitis C virus antibody positive	4
Hepatitis B surface antigen positive	3
NASH/NAFLD	2
Unidentified	2
Prior treatment of C-ion RT	
TACE	6
TACE and RFA	2
TACE and hepatic artery infusion chemotherapy	2
Treatment naïve	1
Site of direct invasion	
Major branch of the portal vein	3
Major branch of the hepatic vein	5
Both major branches of the portal vein and the hepatic vein	3
Child-Pugh class	
A:B	10:1
Barcelona Clinic Liver Classification Stage	
A:B:C	2:0:9
Albumin-Bilirubin Grade	
1:2a:2b:3	3:2:5:1
Pretreatment AFP, IU/ml	
< 200	6
200–400	2
> 400	3
Indocyanine green retention rate at 15 min	
< 15%	5
15–30%	4
> 30%	2
Median (range)	15.9 (4.9–108.7)

^†^*Abbreviations: AFP* alpha-fetoprotein, *C-ion RT* carbon ion radiotherapy, *NASH/NAFLD* non-alcoholic fatty liver disease/non-alcoholic steatohepatitis, *RFA* percutaneous radiofrequency ablation, *TACE* transarterial chemoembolization

### Clinical outcomes

**Fig. 2:**
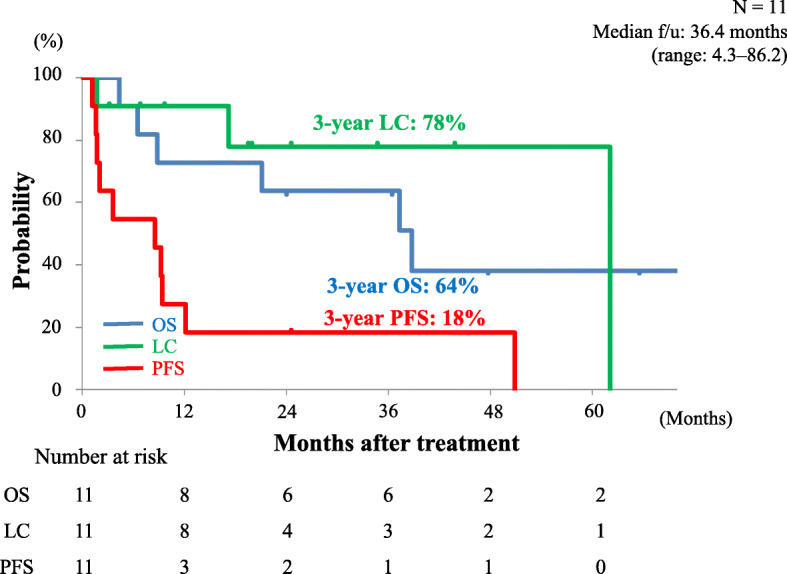
Kaplan-Meier curves: overall survival (blue line), local recurrence (green line), and progression-free survival (red line) in all patients. Number at risk is shown below the Fig. OS, overall survival; LC, local control; PFS, progression-free survival; f/u, follow-up

We calculated probabilities of OS, LC, and PFS rates and determined the recurrence pattern. The OS, LC, and PFS curves of all the patients are shown in Fig. 2. The 3-year estimated OS, LC, and PFS rates were 64, 78, and 18%, respectively. At the time of analysis, recurrence after C-ion RT was observed in 10 patients; 1 patient had local recurrence, 1 had both local recurrence and intrahepatic recurrence outside of the target region, 1 had local recurrence after distant metastases to the lung, 6 had intrahepatic recurrence outside the target region, and 1 had intrahepatic recurrence outside the target region after distant metastases to the lung. The details of treatment after recurrence are summarized in Table 2. A total of six patients died of HCC and one patient died of rectal cancer.

**Table 2:** Treatment after recurrence

Case number	PFS duration (months)	First site of recurrence	Number of treatments after recurrence					
			Surgery	C-ion RT	RFA	TACE	TAI	Molecular targeted therapy
1	1.7	Intrahepatic recurrence	None	None	None	2	None	None
2^a^	50.9	Distant metastases (lung)	None	1	None	None	None	1 (Lenvatinib)
3	9.5	Intrahepatic recurrence	None	None	None	1	None	1 (Sorafenib)
4	24.7	No recurrence	None	None	None	None	None	None
5	9.3	Intrahepatic recurrence	1	None	1	3	None	2 (Sorafenib and Lenvatinib)
6^b^	12.2	Distant metastases (lung)	1	1	1	None	1	None
7	3.6	Intrahepatic recurrence	None	None	1	None	None	1 (Sorafenib)
8	8.6	Intrahepatic recurrence	None	None	1	None	None	1 (Sorafenib)
9	1.2	Intrahepatic recurrence	None	None	None	1	None	1 (Lenvatinib)
10	2.1	Intrahepatic recurrence	None	None	None	1^c^	None	None
11	1.8	Local recurrence	None	None	None	None	None	None

^a^Patient in Case 2 had local recurrence after distant metastases to the lung and received C-ion RT for local recurrence as a re-irradiation

^b^Patient in Case 6 had intrahepatic recurrence outside the target region after distant metastases to the lung, who received surgery for the lung metastases and C-ion RT for the intrahepatic recurrence

^c^Patient in Case 10 received transarterial embolization

^*¶*^*Abbreviations: C-ion RT* carbon ion radiotherapy, *PFS* progression-free survival, *RFA* percutaneous radiofrequency ablation, *TACE* transarterial chemoembolization, *TAI* transcatheter arterial infusion chemotherapy

**Fig. 3:**
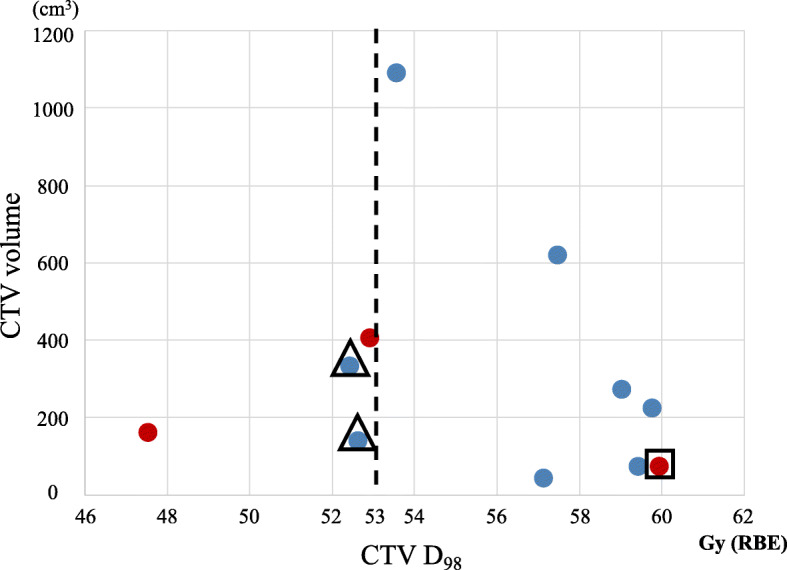
Scatterplots of the CTV volume, CTV D98, and presence or absence of local recurrence. Blue circles indicate tumor control cases and red circles indicate tumor recurrence cases. A red circle surrounded by a square indicates a case of local recurrence more than five years after C-ion RT, and blue circles surrounded by a triangle indicate cases of prescribed dose of 52.8 Gy (RBE) of C-ion RT. CTV, clinical target volume; RBE, relative biological effectiveness

In order to to identify the dosimetric parameters associated with local control after C-ion RT, we then performed a dose–volume analysis. The median CTV volume and CTV D_98_ on DVH analysis were 227 cm^3^ (range: 76–1090) and 57.1 Gy (RBE) (range: 47.5–59.9), respectively. Scatterplots of the CTV volume, CTV D_98_, and presence or absence of local recurrence are shown in Fig. 3. These plots revealed that patients with higher D_98_ tended to have locally controlled tumor regardless of CTV volume. One patient with high CTV D_98_ (red circle surrounded by a square) had local recurrence more than 5 years after the treatment with C-ion RT. Two patients with locally controlled tumors and low CTV D_98_ (blue circles surrounded by a triangle) were prescribed C-ion RT at a dose of 52.8 Gy (RBE). In the other two patients with a locally recurrent tumor and low CTV D_98_ (red circles in lower than 53 Gy [RBE] area), CTV D_98_ was lowered due to the priority given to the dose constraint over the GI tract. Patients with higher CTV D_98_ tended to have no local recurrence or long-term local control after C-ion RT.

### Toxicities

All details of the observed acute and late toxicities are listed in Table 3. No patient developed radiation-induced liver diseases, or grade 2 or higher toxicities in the acute and late phases. Two of 11 patients with Child-Pugh class A progressed to class B within 3 months after treatment with C-ion RT. After 3 months from the initiation of C-ion RT, 3 of 10 patients with Child-Pugh class A progressed to class B. No severe toxicities had developed in our study patients.

**Table 3:** Acute and late toxicities graded by CTCAE, version 4.0 (*N =* 11)

	Grade 0	Grade 1	Grade 2	Grade 3	Grade 4
**Acute toxicities**					
Dermatitis	2	9	0	0	0
GI tract	11	0	0	0	0
Pneumonitis	8	3	0	0	0
Encephalopathy	11	0	0	0	0
Ascites	9	2	0	0	0
**Late toxicities**					
Dermatitis	3	8	0	0	0
GI tract	11	0	0	0	0
Pneumonitis	9	2	0	0	0
Encephalopathy	11	0	0	0	0
Ascites	9	2	0	0	0
Bone fracture	11	0	0	1	0

^†^*Abbreviations: CTCAE* Common Terminology Criteria for Adverse Events, *GI* gastrointestinal tract

## Discussion

The current study demonstrated that C-ion RT showed favorable clinical outcomes in patients with LAHCC. In our study, the 3-year estimated OS, LC, and PFS rates were 64, 78, and 18%, respectively, with minimal toxicities. A previous study on C-ion RT outcomes in patients with HCC in a multi-institutional analysis, which did not include locally advanced cases, showed that 3-year LC rate was 81% [9]. The result of LC shown in that study was similar to that in our study, although all patients analyzed had locally advanced disease cases. For various LAHCC treatments, the median OS in molecular targeted therapy ranged between 5.3 and 11.5 months [3–5, 21], while that in hepatic arterial infusion chemotherapy with radiotherapy was 9.9 months [25], and that in TACE-based multidisciplinary treatment ranged from 7.0 to 12.7 months [5, 26]; the 3-year OS rates in surgery based multidisciplinary treatment ranged from 13 to 68% [15, 27, 28]. Additionally, Komatsu et al. reported on the comparison of clinical outcomes between LAHCC treated with particle therapy and liver resection in a matched-pair analysis [29]. Clinical outcomes of these other anti-cancer treatments are summarized in Table 4. They concluded that particle therapy was potentially preferable over liver resection in LAHCC. Although another anticancer therapy for LAHCC showed a wide range of outcomes in OS, the 3-year OS of 64% for C-ion RT-based multidisciplinary treatment shown in our study, appears to be comparable or favorable. Therefore, we propose that C-ion RT could be one of the therapy options for the multidisciplinary treatment of LAHCC.

**Table 4:** Comparison between the present study and the previous studies of LAHCC

Reference	Year	n	Treatment Method	OS
Kudo M, et al. [3]	2018	250	Lenvatinib	Median OS: 11.5 months
Bruix J, et al. [4]	2012	108	Sorafenib	Median OS: 8.1 months
Yoon SM, et al. [5])	2018	45	Sorafenib	Median OS: 9.9 months
Kodama K, et al. [21]	2018	36	Sorafenib	Median OS: 5.3 months
Kodama K, et al. [21]	2018	36	HAIC with RT	Median OS: 9.9 months
Yoon SM, et al. [5]	2018	45	TACE with RT	Median OS: 12.7 months
Zhang X, et al.^a^ [22]	2017	21–604	Sorafenib	Median OS: 7.0–13.0 months
Kudo M, et al. [15]	2019	1101	Surgery	3y-OS: 40%
Liu PH, et al. [23]	2014	247	Surgery	3y-OS: 68%
Shi J, et al. [24]	2010	406	Surgery	3y-OS: 13%
Komatsu S, et al. [25]	2017	19	Particle RT (proton beam therapy and C-ion RT)	Median OS: 24.6 months
Present study		11	C-ion RT	Median OS: 36.4 months, 3y-OS: 64%

^a^Review article

^†^*Abbreviations: C-ion RT* carbon ion radiotherapy, *HAIC* hepatic arterial infusion chemotherapy, *OS* overall survival, *RT* radiotherapy, *TACE* transarterial chemoembolization, *3y-OS* 3-year overall survival

The results of our study showed that patients with higher D_98_ for CTV tended to have locally controlled tumors, including local recurrence of more than 5 years after C-ion RT (Fig. 3). Indeed, two patients with locally controlled tumors and low CTV D_98_ who were prescribed a dose of 52.8 Gy (RBE), and all patients with CTV D_98_ who received more than 53 Gy (RBE), had no local recurrence with long-term local control after C-ion RT. This result suggested that high-dose C-ion beam administration can achieve local control, which may result in long-term local recurrence-free survival. Previous studies compared DVH for tumorous and normal liver between C-ion RT and X-ray RT (SBRT and IMRT) [10, 11]. Particularly for LAHCC, which is a large tumor or/and a tumor with irregular shapes, the dose required for the normal liver may be higher than that used for HCC, which has no macroscopic vascular invasion. Higher doses delivered to the normal liver resulted in a higher risk of radiation-induced liver disease [30]; the prescribed dose must therefore be decreased to avoid development of radiation-induced liver disease. It is therefore difficult to administer sufficient tumor control doses for LAHCC with X-ray RT. In contrast, C-ion RT can decrease the dose delivered to the healthy liver while administering a sufficient dose to the tumor, due to its higher achievable dose concentration owing to the sharp lateral penumbra and distal tail-off.

Yoon et al. showed that TACE combined with X-ray RT resulted in improved prognosis compared with molecular targeted therapy alone [5]. In terms of dose distribution, C-ion RT showed higher dose concentrations than X-ray RT [10, 11]; therefore, C-ion RT can result in reduced dose distribution to the healthy liver region without reducing the dose delivered to the tumor, thereby preserving liver function. If liver function can be preserved, the number of treatment options for preventing HCC recurrence may be increased. Liver function preservation is crucial for HCC patients who may need repeat treatment because of frequent recurrences, such as in cases of LAHCC. In our study, nine patients needed multiple treatments for recurrent tumors (Table 2), and it may be possible that liver function preservation after C-ion RT enabled multiple treatment rounds after recurrence. Therefore, C-ion RT offers the advantage of liver function preservation during HCC treatment compared with X-ray RT, and TACE combined with C-ion RT may confer better prognosis than TACE combined with X-ray RT.

Proton beam therapy may be one of the treatment options for LAHCC in multidisciplinary treatment because of its higher dose concentration compared to X-ray RT [29, 31]. In terms of the dose fractionation schedule, proton beam therapy needs 8–38 fractions depending on the tumor location. In contrast, C-ion RT needs only 4 or 12 fractions. When combined with other anti-cancer therapies in multidisciplinary treatment, a shorter dose fractionation schedule offers an advantage in terms of the overall treatment time for planned sequential treatment. and may therefore improve the prognosis.

Our study had several limitations. First, this study was a single-institution retrospective analysis with a small number of patients. Second, there were a small number of patients with the most advanced stage of HCC involving a major branch of the portal or hepatic vein, such as the Vp4 and Vv3. Therefore, clinical outcomes observed here may have appeared favorable. Third, only the patients who were likely to benefit from local treatment were analyzed in the current study. The other reports of anti-cancer treatment for LAHCC included patients in whom systemic therapy was indicated, with little scope for local treatment; this patient bias may have affected survival rates.

## Conclusions

Although LAHCC remains difficult to cure, C-ion RT-based multidisciplinary treatment showed favorable clinical outcomes with a high rate of local control and minimal toxicity. This finding suggests that C-ion RT may be a useful treatment option in the multidisciplinary therapy of LAHCC in patients who are likely to benefit from local treatment.

## Data Availability

The datasets generated and/or analyzed during the current study are not publicly available because it contains personal information, but are available from the corresponding author on reasonable request.

## Abbreviations

C-ion RT: Carbon ion radiotherapy
CT: Computed tomography
DVH: Dose–volume histogram
GTV: Gross tumor volume
HCC: Hepatocellular carcinoma
IMRT: Intensity-modulated radiotherapy
ITV: Internal target volume
LAHCC: Locally advanced HCC
LC: Local control
MRI: Magnetic resonance imaging
OS: Overall survival
PFS: Progression-free survival
PTV: Planning target volume
RFA: Radiofrequency ablation
RBE: Relative biological effectiveness
RT: Radiotherapy
SBRT: Stereotactic body radiotherapy
TACE: Transarterial chemoembolization

## References

[1] EASL-EORTC clinical practice guidelines: management of hepatocellular carcinoma. J Hepatol. 2012;56:908–43. 10.1016/j.jhep.2011.12.001 22424438

[2] Llovet JM, Ricci S, Mazzaferro V, Hilgard P, Gane E, Blanc JF (2008). Sorafenib in advanced hepatocellular carcinoma. N Engl J Med.

[3] Kudo M, Finn RS, Qin S, Han KH, Ikeda K, Piscaglia F (2018). Lenvatinib versus sorafenib in first-line treatment of patients with unresectable hepatocellular carcinoma: a randomised phase 3 non-inferiority trial. Lancet..

[4] Bruix J, Raoul JL, Sherman M, Mazzaferro V, Bolondi L, Craxi A (2012). Efficacy and safety of sorafenib in patients with advanced hepatocellular carcinoma: subanalyses of a phase III trial. J Hepatol.

[5] Yoon SM, Ryoo BY, Lee SJ, Kim JH, Shin JH, An JH (2018). Efficacy and safety of transarterial chemoembolization plus external beam radiotherapy vs sorafenib in hepatocellular carcinoma with macroscopic vascular invasion: a randomized clinical trial. JAMA Oncol.

[6] Kasuya G, Kato H, Yasuda S, Tsuji H, Yamada S, Haruyama Y (2017). Progressive hypofractionated carbon-ion radiotherapy for hepatocellular carcinoma: combined analyses of 2 prospective trials. Cancer..

[7] Shiba S, Abe T, Shibuya K, Katoh H, Koyama Y, Shimada H (2017). Carbon ion radiotherapy for 80 years or older patients with hepatocellular carcinoma. BMC Cancer.

[8] Shibuya K, Ohno T, Katoh H, Okamoto M, Shiba S, Koyama Y (2019). A feasibility study of high-dose hypofractionated carbon ion radiation therapy using four fractions for localized hepatocellular carcinoma measuring 3cm or larger. Radiother Oncol.

[9] Shibuya K, Ohno T, Terashima K, Toyama S, Yasuda S, Tsuji H (2018). Short-course carbon-ion radiotherapy for hepatocellular carcinoma: a multi-institutional retrospective study. Liver Int.

[10] Shiba S, Shibuya K, Kawashima M, Okano N, Kaminuma T, Okamoto M (2020). Evaluation of advantages in dose-distribution with carbon ion radiotherapy versus intensity-modulated radiation therapy for hepatocellular carcinoma with macroscopic vascular invasion. Anticancer Res.

[11] Abe T, Saitoh J, Kobayashi D, Shibuya K, Koyama Y, Shimada H (2015). Dosimetric comparison of carbon ion radiotherapy and stereotactic body radiotherapy with photon beams for the treatment of hepatocellular carcinoma. Radiat Oncol.

[12] Tsujii H, Kamada T, Shirai T, et al. Carbon-Ion Radiotherapy: Springer; 2014.

[13] Nakano T, Suzuki Y, Ohno T, Kato S, Suzuki M, Morita S (2006). Carbon beam therapy overcomes the radiation resistance of uterine cervical cancer originating from hypoxia. Clin Cancer Res.

[14] Cui X, Oonishi K, Tsujii H, Yasuda T, Matsumoto Y, Furusawa Y (2011). Effects of carbon ion beam on putative colon cancer stem cells and its comparison with X-rays. Cancer Res.

[15] Kudo M, Izumi N, Kubo S, Kokudo N, Sakamoto M, Shiina S (2020). Report of the 20th Nationwide follow-up survey of primary liver Cancer in Japan. Hepatol Res.

[16] Llovet JM, Bru C, Bruix J (1999). Prognosis of hepatocellular carcinoma: the BCLC staging classification. Semin Liver Dis.

[17] Johnson PJ, Berhane S, Kagebayashi C, Satomura S, Teng M, Reeves HL (2015). Assessment of liver function in patients with hepatocellular carcinoma: a new evidence-based approach-the ALBI grade. J Clin Oncol.

[18] Kanematsu N (2011). Dose calculation algorithm of fast fine-heterogeneity correction for heavy charged particle radiotherapy. Phys Med.

[19] Inaniwa T, Kanematsu N, Matsufuji N, Kanai T, Shirai T, Noda K (2015). Reformulation of a clinical-dose system for carbon-ion radiotherapy treatment planning at the National Institute of Radiological Sciences, Japan. Phys Med Biol.

[20] Tashiro T, Ishii T, Koya J, Okada R, Kurosawa Y, Arai K (2013). Technical approach to individualized respiratory-gated carbon-ion therapy for mobile organs. Radiol Phys Technol.

[21] Kubota Y, Tashiro M, Shinohara A, Abe S, Souda S, Okada R (2015). Development of an automatic evaluation method for patient positioning error. J Appl Clin Med Phys.

[22] Abe S, Kubota Y, Shibuya K, Koyama Y, Abe T, Ohno T (2017). Fiducial marker matching versus vertebral body matching. Dosimetric impact of patient positioning in carbon ion radiotherapy for primary hepatic cancer. Phys Med.

[23] Kubota Y, Katoh H, Shibuya K, Shiba S, Abe S, Sakai M (2019). Comparison between bone matching and marker matching for evaluation of intra- and inter-fractional changes in accumulated of carbon ion radiotherapy for hepatocellular carcinoma. Radiother Oncol.

[24] US Department of Health and Human Services. Common Terminology Criteria for Adverse Events (CTCAE) Version 4.0. In: National Institutes of Health: National Cancer Institute; 2009.

[25] Kodama K, Kawaoka T, Aikata H, Uchikawa S, Nishida Y, Inagaki Y (2018). Comparison of outcome of hepatic arterial infusion chemotherapy combined with radiotherapy and sorafenib for advanced hepatocellular carcinoma patients with major portal vein tumor thrombosis. Oncology..

[26] Zhang X, Wang K, Wang M, Yang G, Ye XF, Wu MC (2017). Transarterial chemoembolization (TACE) combined with sorafenib versus TACE for hepatocellular carcinoma with portal vein tumor thrombus: a systematic review and meta-analysis. Oncotarget..

[27] Liu PH, Lee YH, Hsia CY, Hsu CY, Huang YH, Chiou YY (2014). Surgical resection versus transarterial chemoembolization for hepatocellular carcinoma with portal vein tumor thrombosis: a propensity score analysis. Ann Surg Oncol.

[28] Shi J, Lai EC, Li N, Guo WX, Xue J, Lau WY (2010). Surgical treatment of hepatocellular carcinoma with portal vein tumor thrombus. Ann Surg Oncol.

[29] Komatsu S, Kido M, Asari S, Toyama H, Ajiki T, Demizu Y (2017). Particle radiotherapy, a novel external radiation therapy, versus liver resection for hepatocellular carcinoma accompanied with inferior vena cava tumor thrombus: a matched-pair analysis. Surgery..

[30] Liang SX, Zhu XD, Xu ZY, Zhu J, Zhao JD, Lu HJ (2006). Radiation-induced liver disease in three-dimensional conformal radiation therapy for primary liver carcinoma: the risk factors and hepatic radiation tolerance. Int J Radiat Oncol Biol Phys.

[31] Komatsu S, Fukumoto T, Demizu Y, Miyawaki D, Terashima K, Niwa Y (2011). The effectiveness of particle radiotherapy for hepatocellular carcinoma associated with inferior vena cava tumor thrombus. J Gastroenterol.

